# The pervasive avoidance of prospective statistical power: major consequences and practical solutions

**DOI:** 10.3389/fpsyg.2015.00726

**Published:** 2015-05-28

**Authors:** Patrizio E. Tressoldi, David Giofré

**Affiliations:** Dipartimento di Psicologia Generale, Università di Padova Padova, Italy

**Keywords:** prospective statistical power, sample size planning, effect size, reproducibility of results, NHST

## The pervasive overlooked importance of prospective statistical power

The estimation of the prospective statistical power (*PSP*) is mandatory when using a classical Neyman-Pearson statistical method that together with the one by Fisher, represents one of the pillars of the so-called frequentist statistical approach (see Perezgonzalez, 2015, for a historical review and a tutorial). At present the Null Hypothesis Significance Testing (NHST) represents the most used statistical approach in many research fields, from psychology to medicine, from neuroscience to ecology. Unfortunately, in the course of the history of their application, these two methods have been mixed adopting the Fisher approach for hypotheses or model comparisons and their differences ignored.

The uncritical application of the NHST statistical approach in ignoring its assumptions, strengths and weakness, has been considered one if not the principal cause of the “crisis of confidence” in scientific evidence (Ioannidis, 2005; Pashler and Wagenmakers, 2012). To counter this serious situation, apart from some explicit declaration to completely abandon the NHST (Wagenmakers et al., 2011; Harlow et al., 2013), many journals and scientific associations have published new statistical guidelines wherein the use of the *PSP* is explicitly required. For example, for psychology, in the last edition of the APA Manual (American Psychological Association, 2010, p. 30), it is clearly recommended “…*When applying inferential statistics, take seriously the statistical power considerations associated with your tests of hypotheses. Such considerations relate to the likelihood of correctly rejecting the tested hypotheses, given a particular alpha level, effect size, and sample size. In that regard, you should routinely provide evidence that your study has sufficient power to detect effects of substantive interest (e.g., see Cohen, 1988). You should be similarly aware of the role played by sample size in cases in which not rejecting the null hypothesis is desirable (i.e., when you wish to argue that there are no differences), when testing various assumptions underlying the statistical model adopted (e.g., normality, homogeneity of variance, homogeneity of regression), and in model fitting.”*

Similarly, the Society for Personality and Social Psychology (SPSP) Task Force on Publication and Research Practices (Funder et al., 2014, p. 3), in his statistical primer and recommendations for improving the dependability of research, declare “….*An important goal in designing research is to maximize statistical power, the probability that the null hypothesis will be rejected if there is, in fact, a true effect of the specified size in the population. However, this goal can be challenging, statistical power will be limited by factors such as sample size, measurement error, and the homogeneity of the participants*. Cohen (1988) *suggested a convention that investigations should normally have power = 0.8 to detect a true effect of the specified size in the population.”*

Almost identical guidelines have been now endorsed by The Psychonomic Society's Publications Committee and Ethics Committee and the Editors in Chief of the Society's six journals “…*Studies with low statistical power produce inherently ambiguous results because they often fail to replicate. Thus it is highly desirable to have ample statistical power and to report an estimate of a priori [prospective] power (not post hoc power [estimated after the study completion]) for tests of your main hypotheses…. the Method section should make clear what criteria were used to determine the sample size. The main points here are to (a) do what you reasonably can to attain adequate power and (b) explain how the number of participants was determined* (Psychonomic Society, 2014).

## A brief survey on the use of *PSP*

The problem of underpowered studies has a long history in psychology (see Maxwell, 2004 for a review), but it seems there have not been any changes up to today. In their survey of statistical reporting practices in psychology Fritz et al. (2013), observed that *PSP* was reported in only 3% of over 6000 articles. Vankov et al. (2014), reported that *PSP*, or at least some mention of statistical power, was observed in only 5% of all 183 empirical articles published in *Psychological Science* in the 2012. Similarly, Tressoldi et al. (2013), in their survey of the statistical reporting practices, observed that *PSP* was reported in less than 3% of the studies published in the 2011 volumes of four journals with very high impact factors, Science, Nature, Nature Neuroscience and Nature Medicine and above 60% in The Lancet and The New England Journal of Medicine (NEJM). This large difference was probably due to the adherence of The Lancet and the NEJM to the (CONsolidated Standards of Reporting Trials) 2010 guideline which explicitly requires disclosing how sample size was determined (Schulz et al., 2010).

Our survey of all original research papers published in Frontiers of Psychology in 2014, revealed that *PSP* or at least a justification on how the sample size was determined, was found in only 2.9% out of 853 eligible studies^1^.

To sum up, it seems very clear that the use and hence the importance of *PSP* continue to be neglected in most empirical studies, independently from the Impact Factor of the journals with exceptions for some medical journals were it is explicitly required in the submission guidelines for Authors. The reason for this state of affair is not the aim of this paper but we endorse Schimmack's (2012, p. 561) interpretation: “*The most probable and banal explanation for ignoring power is poor statistical training at the undergraduate and graduate levels,”* with all consequences emerging when those people act as reviewers or Editors.

## Consequences

What are the consequences of this overlooked use of *PSP* on the credibility of scientific findings? Are they trivial as those related to the reporting of exact vs. approximate *p* values or the use of standard error instead of confidence intervals as error bars?

Button et al. (2013), estimated that the median statistical power of 48 meta-analyses of neuroscience articles published in 2011, comprising 730 studies, was equal to 0.21. For psychological studies, the survey by Bakker et al. (2012) on 281 primary studies indicated an average power of about 0.35, meaning that the typical psychological study has slightly more than a one-in-three chance of finding an effect if it does exist.

The dramatic consequence of this underpowered situation in most of published studies is an overestimation of effect size and a low reproducibility of the scientific findings given the low probability of observing the same results. To obtain a measure of the replicability of empirical studies based on an estimate of their statistical power, Ulrich Schimmak has devised the R-Index available here: . Simple simulations with this software, will clarify the relationship between the statistical power and the level of replicability.

## Remediation

We think that the remediation of this state of affairs requires the contribution of both the editors of the scientific journals and of all authors of scientific investigations.

### The editors of scientific journals

In our opinion a mandatory requirement to disclose how the sample(s) size was determined in all experimental studies might be an almost definite solution to this problem.

This requirement should be made clear in the authors' submission guidelines of all scientific journals and endorsed by all their editors in chief. The outcomes of this policy are already visible in some medical journals like The Lancet and the NEJM where it has already been applied.

The impact of analogous recommendations in documents from scientific associations, like the APA, seems ineffective in changing the statistical practices of authors even when they submit their paper to the journals published by these scientific associations.

### All authors

The first requirement is to be aware of the critical importance of how to define the size of the sample(s) to be used in the experimental investigations and how serious the consequences are for their scientific results and science in general when neglecting this fact.

The availability of freeware software, running both for Windows and Mac operating systems and online calculators for estimating the sample(s) size necessary to achieve the desired *PSP*, should facilitate the implementation of this practice. In our opinion, the first choice is G^*^Power (Faul et al., 2007; http://www.gpower.hhu.de), followed by the online calculators available here http://powerandsamplesize.com and http://jakewestfall.org/pangea. For more complex experimental design, for example *PSP* with crossed random effects, see Westfall et al. (2014) and their online calculator available on http://jakewestfall.org/power.

### And when there are difficulties in recruiting the necessary sample(s) size?

Given that *PSP* also depends on the number of comparisons being performed and the size of the effects being studied, when the number of comparisons is high and/or the size of the effects are low, for example below 0.20 in standard units, the size of the sample(s) necessary to achieve a *PSP* of at least 0.80 may be very high, making it very difficult to investigate some phenomena. For example to achieve a *PSP* of 0.80 estimating a standardized effect size of 0.20 for two independent groups comparison, a total of 620 participants are needed.

Here follows some practical solutions to this problem.

A first solution could be a collaborative multisite study with other researchers interested in the investigation of the same phenomena.

Another solution could be to find ways to reduce the size of the sample(s). For example, Lakens (2014) suggested how to obtain high−powered studies efficiently using *sequential analyses* to reduce the sample size of studies by 30% or more by controlling for the Type 1 error and the questionable research practice of “optional stopping” (John et al., 2012).

Among other proposals, Perugini et al. (2014) suggest to use the “*safeguard power analysis*,” which uses the uncertainty in the estimate of the effect size to achieve a better likelihood of correctly identifying the population effect size. Vanbrabant et al. (2015), offer sample-size tables for ANOVA and regression when using Constrained statistical inference.

A more radical solution is that of not using the *PSP* and its statistical postulates at all, but rather adopting other statistical approaches. Schimmack (2012) for example, suggested publishing studies with significant and nonsignificant results ignoring *p* values altogether and to focus more on effect sizes and their estimation by using confidence intervals in line with the so called “statistical reform” movement endorsed recently by the editor of Psychological Science (Eich, 2014) and the ban of the NHST adopted by Trafimow and Marks (2015) for all submission to the Basic and Applied Social Psychology journal. Similarly, Gelman and Carlin (2014) suggested to focus on estimates and uncertainties rather than on statistical significance. All these parameter estimations and effect sizes can be used both for simulations and meta-analyses, fostering what Cumming (2012) and others defined “meta-analytic thinking.” See: “*shifting the question from whether or not a single study provided evidential weight for a phenomenon to the question of how well all studies conducted thus far support conclusions in regards to a phenomenon of interest* (Braver et al., 2014, p. 334).”

Shifting from the NHST to a Bayesian statistical approach, it is possible to supplement the statistical analyses by calculating the Bayes Factor for model comparisons of interest, demonstrating how it is possible for low-power experiments to yield strong evidence, and for high-power experiments to yield weak evidence as suggested by Wagenmakers et al. (2014). Furthermore, if we consider that a Bayesian hypothesis testing approach is immune to the dangers of the “optional stopping” research practice when using the classical NHST approach (Sanborn and Hills, 2014), this renders this proposal very practical and attractive.

## Final remarks

*PSP* cannot continue to be ignored nor its consequences on the credibility of scientific evidence. Practical solutions are at hand and hence their implementations call forth the responsibility of all scientists.

### Conflict of interest statement

The authors declare that the research was conducted in the absence of any commercial or financial relationships that could be construed as a potential conflict of interest.

## Footnotes

^1^Excluding theoretical, qualitative, single-case, simulation studies, and meta-analyses. Raw data are available on http://figshare.com/articles/Prospective_Statistical_Power_in_Frontiers_in_Psychology_2014/1304144.
